# SCA27B in Brazil: frequency, phenotype and genotype–phenotype correlations

**DOI:** 10.1007/s00415-026-13880-4

**Published:** 2026-05-21

**Authors:** Amanda de Jesus Araujo Dias, Cynthia Silveira, Adriana Mendes Vinagre, Luciana Cardoso Bonadia, Nadson Bruno Serra Santos, Thiago Junqueira R. Rezende, Luiza Alves Corazza, José Luiz Pedroso, Orlando Graziani P. Barsottini, Fabricio Diniz de Lima, Marcondes C. França Junior

**Affiliations:** 1 Department of Neurology, School of Medical Sciences, University of Campinas (UNICAMP), Rua Tessália Vieira de Camargo, 126, Cidade Universitária “Zeferino Vaz”, Campinas, São Paulo, 13083-887 Brazil; 2 Laboratory of Molecular Genetics, School of Medical Sciences, University of Campinas (UNICAMP), Campinas, São Paulo, Brazil; 3 Department of Neurology, Ataxia Unit, Federal University of São Paulo (UNIFESP), São Paulo, SP Brazil

**Keywords:** Ataxia, SCA27B, repeat expansion disease, epidemiology

## Abstract

**Background:**

Spinocerebellar Ataxia 27B (SCA27B) is a recently described autosomal dominant ataxia caused by uniallelic GAA intronic expansions at *FGF14*. It is a frequent SCA subtype in North American/European populations, accounting for > 20% of all SCAs in some series. Despite that, its frequency as well as phenotype in Latin America remains to be established.

**Objectives:**

To determine the frequency and the clinical phenotype of SCA27B in a large Brazilian SCA cohort.

**Methods:**

We recruited 498 SCA patients from 322 unrelated families followed in a reference center. All patients had demographic and clinical data collected. The estimated disease progression rate was computed as the ratio between the Scale for the Assessment and Rating of Ataxia (SARA) score and disease duration (in years). Genetic testing included long-range and triplet-primed PCR-based approaches to diagnose SCA1, 2, 3, 6, 7 and SCA27B.

**Results:**

SCA27B was identified in 9 out of the 322 index-patients, totaling 2.8% of all cases. It stands as the fifth most common SCA, surpassed by SCAs 3, 1, 2, and 7, respectively. The typical phenotype in our cases was similar to previous descriptions: late onset (mean age 55.5 years), slow progression (1.0 points/year) and relatively pure ataxic phenotype (11/12). In this cohort, the estimated disease progression rate did correlate with age at onset, but not with (GAA)n.

**Discussion:**

SCA27B is a prevalent SCA in Brazil, but the relative frequency seems to be smaller than in Europe/Canada. It should be included in SCA routine diagnostic protocols. Age at onset might be a potential prognostic marker in this condition.

**Supplementary Information:**

The online version contains supplementary material available at 10.1007/s00415-026-13880-4.

## Introduction

Spinocerebellar ataxias (SCAs) are a clinically and genetically heterogeneous group of monogenic diseases that share progressive cerebellar ataxia and an autosomal dominant mode of transmission as the core features. Affected patients typically present with balance problems, gait instability, dysarthria and oculomotor abnormalities [1, 2]. The prevalence of SCAs is estimated to be approximately 4 per 100.000 individuals [3]. There are currently more than 44 recognized SCA subtypes. Each one is defined according to the underlying gene or mutation associated with it [3]. SCAs 1, 2, 3, 6, 7 and 17 all share an underlying common mechanism—abnormal (CAG) expansions within coding regions of specific genes [2]. This results in the production of an abnormally long chain of glutamine residues in the encoded proteins (for this reason, they are known as Poly-Q SCAs) [4]. Therefore, protein misfolding takes place and triggers a pathological cascade that results in widespread neurodegeneration [5]. Poly-Q SCAs represent the most frequent subtypes worldwide [6]. In countries such as Portugal, Brazil and China, SCA3 is the leading SCA subtype accounting for more than 50% of all cases [7–9].

During the past 20 years much has been learned about the genetic bases of SCAs, but a reasonable number of cases—around 20–30%—remained unsolved from a genetic perspective. A breakthrough came in 2023 when Pellerin et al. discovered a new SCA subtype (SCA27B) in French-Canadian families [10]. It is caused by GAA expansions > 250 within the 1st intron of the *FGF14* gene [10, 11]. SCA27B is characterized by late onset and slowly progressive course often with episodic features and downbeat nystagmus. Since then, SCA27B has emerged as one of the most prevalent SCAs in European and North American populations [10]. In some Canadian reports, it accounts for more than 50% of unsolved SCAs [10]. Despite that, there is limited information on SCA27B beyond purely Caucasian cohorts [10]. This is the case in Latin America, a region where the population is characterized by remarkable genetic admixture with major contributions not only from Europeans, but also from Native Americans and Africans [12]. Brazilian patients with SCA27B were recently reported in a small cohort, but the contribution of the disease relative to other Poly-Q SCAs is not yet clear [13]. Furthermore, one still needs to characterize the phenotype of patients with SCA27B in Brazil. The current study was designed to address these 2 points. To accomplish these goals, we assessed a large cohort of well-characterized SCA patients from a single Brazilian center who underwent genetic testing for both poly-Q SCAs and SCA27B.

## Methods

### Study design and subjects’ selection

**Fig. 1:**
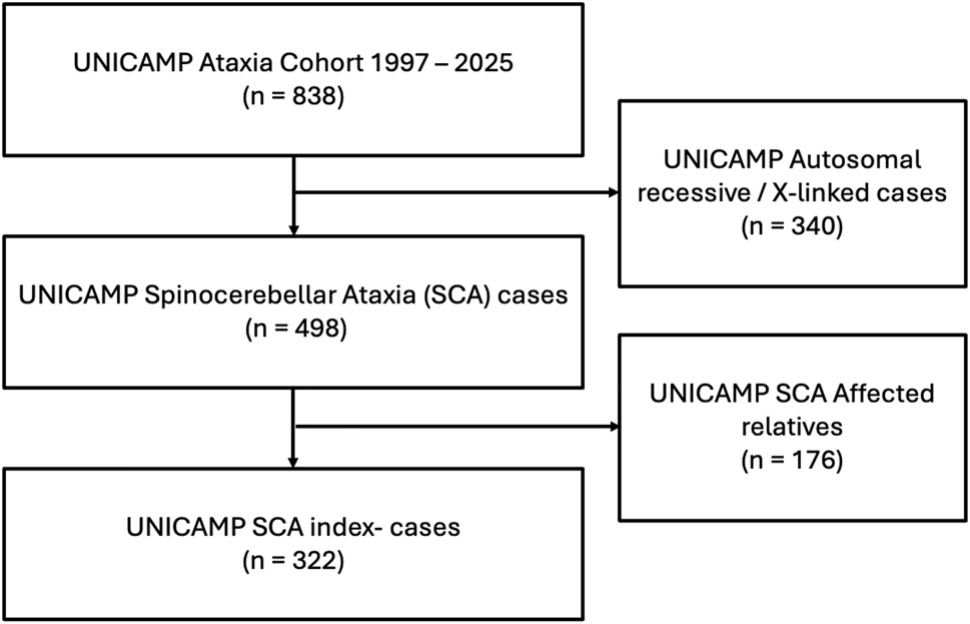
Study flowchart for selection of subjects

This cross-sectional, descriptive study was conducted at the University of Campinas (UNICAMP) between January 2023 and February 2025. We retrieved the medical and genetic data of all patients referred to our center with clinical suspicion of SCA from 1997 to 2025. For enrollment purposes, SCA was defined whenever we had: (a) adult-onset disease; (b) slowly progressive cerebellar ataxia; and (c) AD inheritance. For each patient, acquired causes of adult-onset ataxia were ruled out: alcohol-related etiology, nutritional deficiencies (eg, vitamin E), demyelinating disease or structural lesions of the cerebellum seen on MRI. Subjects with a typical autosomal recessive inheritance pattern and/or with early-onset congenital ataxia were excluded as well (Fig. 1).

At UNICAMP, all patients with suspected SCA are tested for SCA1, 2, 3, 6 and 7 on a routine basis. This is done using long-range PCR protocols with primer sequences and amplification protocols described elsewhere [10]. We identified those patients with SCA who tested negative for the Poly-Q ataxias. These individuals then underwent genetic testing for SCA27B as detailed below.

This study was approved by UNICAMP Research Ethics Committee (CAAE: 83,241,318.3.1001.5404) and all patients signed an Informed Consent Form (ICF) prior to any study-related procedure.

### Clinical data

For all patients who tested positive for SCA27B, demographic and clinical data were collected, including age, sex, family history, onset of symptoms, presence or absence of oculomotor abnormalities, with special attention to downbeat nystagmus. The Scale for Rating and Assessment of Ataxia (SARA) was administered at the baseline visit to quantify ataxia severity. Estimated disease progression rate was then computed as the ratio between the SARA score and disease duration (in years).

### SCA27B testing

Genomic DNA was obtained from peripheral blood lymphocytes using the phenol–chloroform method [14]. SCA27B testing was implemented in our lab following the protocol reported by Pellerin et al. [10]. We used a 2-step approach to search for intronic GAA expansions at *FGF14*. Long-range PCR (LR-PCR) was initially performed to amplify large genomic regions, such as complete genes or genomic regions spanning thousands of base pairs (the primer sequences and experimental conditions are presented in the Supplemental Table 1). LR-PCR products were submitted to capillary electrophoresis in an ABI3500 Genetic Analyzer device and the results were analyzed using the Thermo Cloud pipeline. Samples that tested positive on LR-PCR were confirmed by triplet-primed PCR (TP-PCR), used to amplify a specific region of a gene in both alleles, aiming to detect the presence of an expansion in the selected locus, using specific oligonucleotides (primers) that bind to the ends of the target region (primer sequences and experimental conditions are presented in the Supplemental Table 1). Tests were considered positive only when the (GAA)_n_ was longer than 250 repeats.

### Statistical analyses

We used descriptive statistics to present demographic, clinical and genetic data. Within the SCA27B group, we explored potential clinical correlates (age at onset, disease progression rate) of (GAA)n at *FGF14* using Spearman coefficients. SYSTAT v13.0 was employed for all analyses. P values < 0.05 were considered significant.

### Data sharing

Data are available upon reasonable request to the corresponding author and pending local ethical authorization.

## Results

### Frequency of SCA27B

**Fig. 2:**
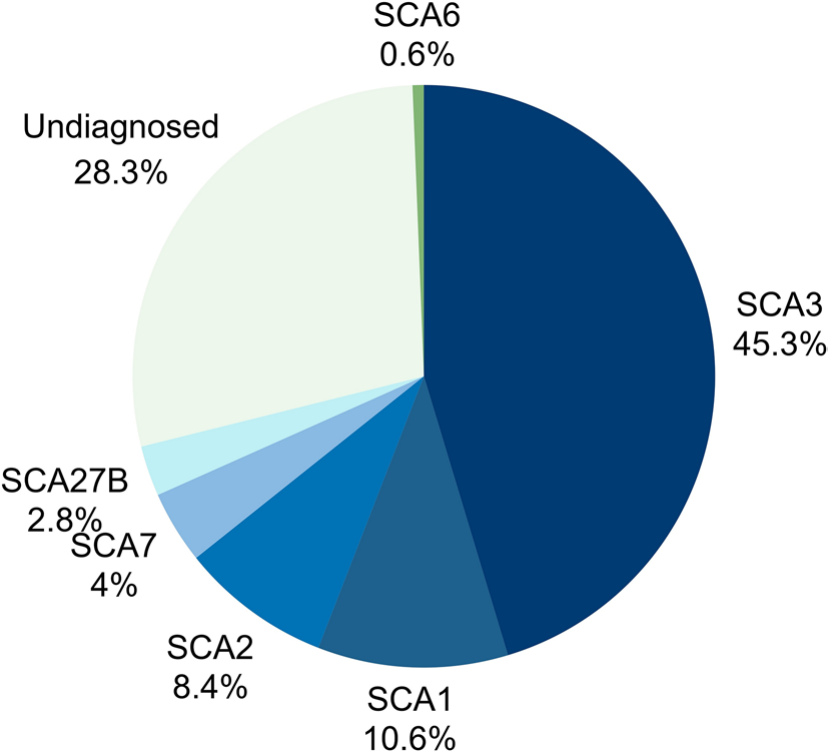
Relative frequency of SCA subtypes in a large Brazilian cohort

**Fig. 3:**
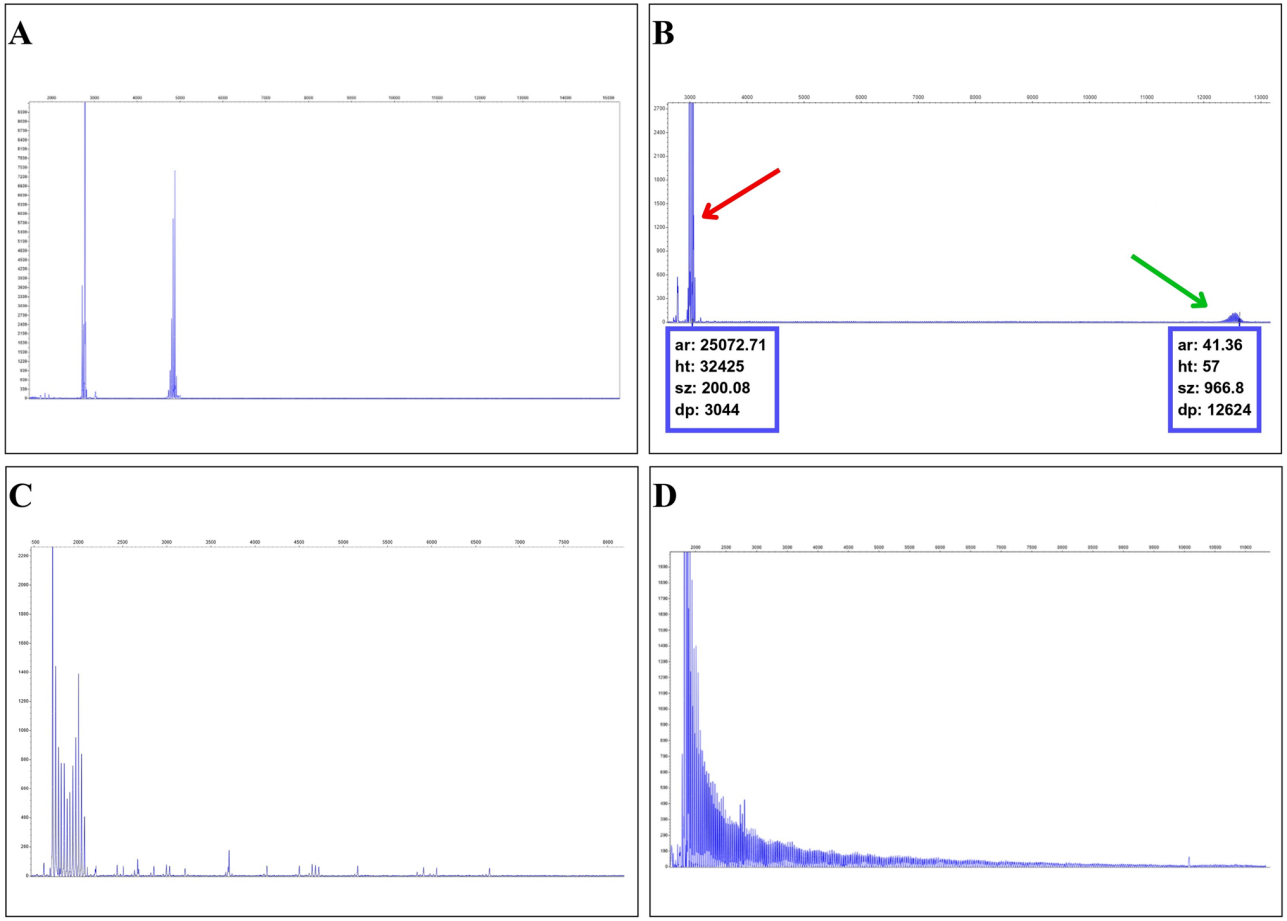
Molecular analysis of GAA expansion in the *FGF14* gene. **A** Electropherogram of capillary electrophoresis analysis of LR-PCR fragments from a negative control. **B** Electropherogram of capillary electrophoresis analysis of LR-PCR fragments from a positive patient. Note that the peak corresponding to the normal-sized allele is marked in red, and the expanded allele is marked in green. **C** Electropherogram of TP-PCR from a negative control for comparison, showing the absence of the expansion pattern. **D** Electropherogram of TP-PCR from the same positive patient, showing the characteristic “sawtooth” pattern that confirms the expansion of the GAA motif. (*bp* base pairs, *LR-PCR* long-range polymerase chain reaction, *TP-PCR* triplet-primed polymerase chain reaction)

We were able to recruit 498 patients with SCA from 322 unrelated families. A causative coding (CAG) expansion in any of the Poly-Q loci was identified in 222 of these families. SCA3 was the most frequent subtype (146/322), followed by SCA1, SCA2, SCA7 and SCA6, respectively (Fig. 2). From the 100 remaining families with unsolved diagnoses, we have found pathogenic intronic (GAA) expansions at *FGF14* in 9 of them. The (GAA) expansion length varied from 251 to 388 (Table 1 and Fig. 3).

**Table 1:** Demographic, genetic, and clinical profile of patients with SCA27B

Patient ID	Sex	Age of onset (years)	Age	Expansion Size	SARA total score	Annual progression rate	Dysarthria	Downbeat Nystagmus	Dysphagia	Diplopia	Pyramidal Signs	Episodic Features
F1	M	72	87	350	28	1.87	x	x	x			x
F2**	F	56	61	315	–	–	x	x	x		x	x
F3	F	63	67	258	4	1		x	x	x		x
F4	M	38	55	269	8	0.47	x		x	x		
F5	M	66	73	315	10	1.43	x	x				
F6	M	44	67	270	12.5	0.54			x			
F6.2	M	56	61	278	2.5	0.50			x			
F7	F	55	66	251	5	0.45						
F7.2	M	51	62	> 375*	7.5	0.68						
F7.3	F	30	53	> 375*	7	0.30		x	x	x		
F7.4	F	45	68	388	2	0.09			x	x		
F8	M	69	79	358	17.5	1.75	x		x			
F8.2	M	41	61	273	8	0.40			x			
F9	M	74	84	258	15.5	1.55	x		x			x

*Expansion confirmed by TP-PCR only; allele size exceeded the detection limit of LR-PCR/capillary electrophoresis

**Patient lost to follow up with unavailable SARA scores. SARA scores were assessed at the last outpatient visit. *M* male, *F* female

### SCA27B phenotype

There were 14 patients with SCA27B from these 9 unrelated families. A detailed phenotypic description of all patients is presented in Table 1.

The age of onset of 1st symptoms (either episodic and/or progressive ataxia) ranged from 38 to 74 years, with a mean of 55.5 ± 13.5 years. The key phenotypic finding was gait ataxia. Downbeat nystagmus was also frequent and observed in 6 individuals. Other signs included dysarthria (5/14 = 35.7%), dysphagia (11/14 = 78.5%), pyramidal signs (1/14 = 7.1%), and diplopia (4/14 = 28.5%). Notably, 42.8% (6/14) of our SCA27B-positive patients manifested postural tremor, a symptom that is very common in SCA27A patients from other cohorts [15]. A distinctive feature in our cases was the relatively low frequency of episodic manifestations (defined as acute onset or worsening of balance complaints over hours/few days), reported only by 4/14 individuals (28.5%). SARA scores ranged from 2 to 28 and the mean estimated annual rate of progression was 1.0. Eight out of the 13 cases had progression rates < 1.0 (61%) (Table 1).

**Fig. 4:**
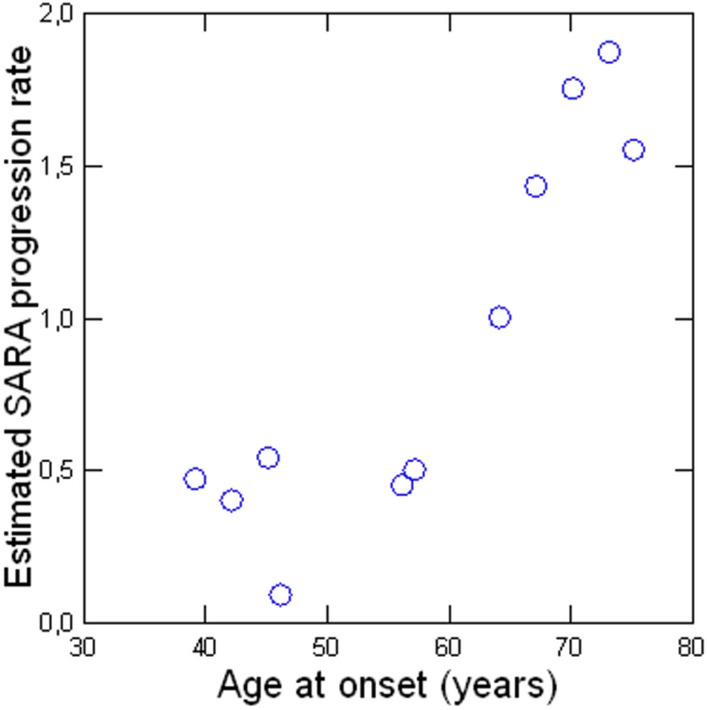
Scatter plot showing the correlation between age at onset and the estimated annual SARA progression rate. Results were computed using information from 13 patients (one of them did not have available SARA information)

Clinico-demographic correlates of (GAA)n were assessed using data from 11 patients because one of them did not have SARA scores (F2) and two were diagnosed based upon TP-PCR, which does not enable precise quantification of the repeat length (repeats were longer than the detection threshold for LR-PCR). In this smaller cohort, (GAA)n did not correlate with SARA scores or the estimated annual SARA progression rate (*p* > 0.05). On the other hand, we were able to investigate whether age at onset would predict disease course using data from 13 patients (only excluding F2). We indeed found a strong positive correlation between age at onset and the estimated annual SARA progression rate (rho = 0.827, *p* < 0.001) (Fig. 4).

## Discussion

In this study, we have validated Novis et al.’s [13] results and confirmed that SCA27B is a prevalent cause of ataxia in the Brazilian population. The original contribution conveyed here is the assessment of the relative frequency of SCA27B compared to other SCAs. This was possible because we managed to implement the protocol for SCA27B testing in our lab. We have indeed shown that SCA27B is the 5th leading cause of SCA: it accounts for 2.8% of SCAs in Brazil. When we consider only unsolved SCAs (after Poly-Q testing), SCA27B is the underlying cause in 9% of patients, figures very close to those reported by Novis et al. (9%). The relative frequency we found in Brazil is smaller than that found in French Canadians (61%) and Europeans (15%), but higher than that reported in Asian populations (1.3%) [10, 16–21]. Furthermore, it seems that SCA27B is not confined to specific Brazilian regions as we have seen unrelated families from at least 3 distinct geographical sites in the country (Northeast, South and Southeast). These patients live far away from Campinas and came to our center to be tested, because we are currently the only center providing genetic testing for SCA27B in the country.

The phenotype of Brazilian patients with SCA27B is similar to previous Canadian and European reports [10]. The age at onset as well as the frequency of downbeat nystagmus and diplopia is in line with Pellerin et al. [10]. Episodic features were slightly less frequent in this series (28.5%), but the reasons for such a difference remain unclear at this point. It is possible that between-group differences on (GAA)_n_ may account for this finding. Indeed, the median GAA repeat length reported in German patients seems to be higher than that found in our cohort (349 v 296.5) [22]. SCA27B is slowly progressive as shown by SARA-based progression rates. The mean value was substantially smaller than that reported in multiple system atrophy (4.62 ± 2.28) and even poly-Q SCAs (range: 0.8–2.1 points/year) [23, 24]. Another interesting finding was the correlation between age at onset and (estimated) disease progression, suggesting this may be an interesting prognostic marker for the disease. We failed to identify correlations between (GAA)n and disease course (either SARA or estimated SARA progression rate). These negative results align with Wilke et al., who had similar findings in their multicentric German cohort [22].

The current report expanded the geographical landscape of SCA27B, but there are some limitations that deserve mention. Even though we were able to recruit a large SCA cohort, this is a single-center study (based in Southeastern Brazil). It is possible that the SCA27B relative frequency might change when we are able to perform more comprehensive surveys including other regions of the country. Furthermore, the SCA27B cohort is relatively small and may have been underpowered to detect genotype vs phenotype correlations. So, additional studies—involving multiple Brazilian centers with a longitudinal design and detailed phenotypic characterization—are needed to better understand the true relevance of the disease in Brazil.

In conclusion, we have shown that SCA27B is a prevalent ataxia in Brazil and should be included in diagnostic algorithms for late-onset ataxia in the country. The search for *FGF14* intronic (GAA) expansions is clinically relevant for diagnostic purposes. Age at onset may be an interesting prognostic marker in this condition.

## Supplementary Information

Below is the link to the electronic supplementary material.

Supplementary file1 (PDF 476 KB)
