# Supplementary material for: SCA27B in Brazil: frequency, phenotype and genotype–phenotype correlations
Source: J Neurol. 2026 May 21;273(6):326. doi: 10.1007/s00415-026-13880-4 (PMC13194261; doi:10.1007/s00415-026-13880-4)
Supplement: Supplementary file 1 — Supplementary file1 (PDF 476 KB) [file 415_2026_13880_MOESM1_ESM.pdf]

SCA27B IN BRAZIL: FREQUENCY, PHENOTYPE AND GENOTYPE-PHENOTYPE CORRELATIONS

Amanda de Jesus Araujo Dias[1], MSc student; Cynthia Silveira[1], PhD; Adriana Mendes Vinagre[1], PhD; Luciana Cardoso Bonadia[2], PhD; Nadson Bruno Serra Santos[1], MD, PhD; Thiago Junqueira R. Rezende, PhD; Luiza Alves Corazza[3], MD; José Luiz Pedroso[3], MD, PhD; Orlando Graziani P. Barsottini[3], MD, PhD; Fabricio Diniz de Lima[1], MD, PhD; Marcondes C. França Junior[1], MD, PhD

[1] Department of Neurology, School of Medical Sciences – University of Campinas (UNICAMP), Campinas, São Paulo, Brazil

[2] Laboratory of Molecular Genetics, School of Medical Sciences – University of Campinas (UNICAMP), Campinas, São Paulo, Brazil

[3] Ataxia Unit, Department of Neurology, Federal University of São Paulo (UNIFESP), São Paulo, SP, Brazil

Address correspondence to:

Marcondes C. França Jr, MD, PhD

Department of Neurology, University of Campinas (UNICAMP)

Rua Tessália Vieira de Camargo, 126. Cidade Universitária "Zeferino Vaz"

Campinas, SP, Brazil - 13083-887

Tel: +55 19 35219217, Fax: +55 19 35217933.

E-mail: mcfjr@unicamp.br

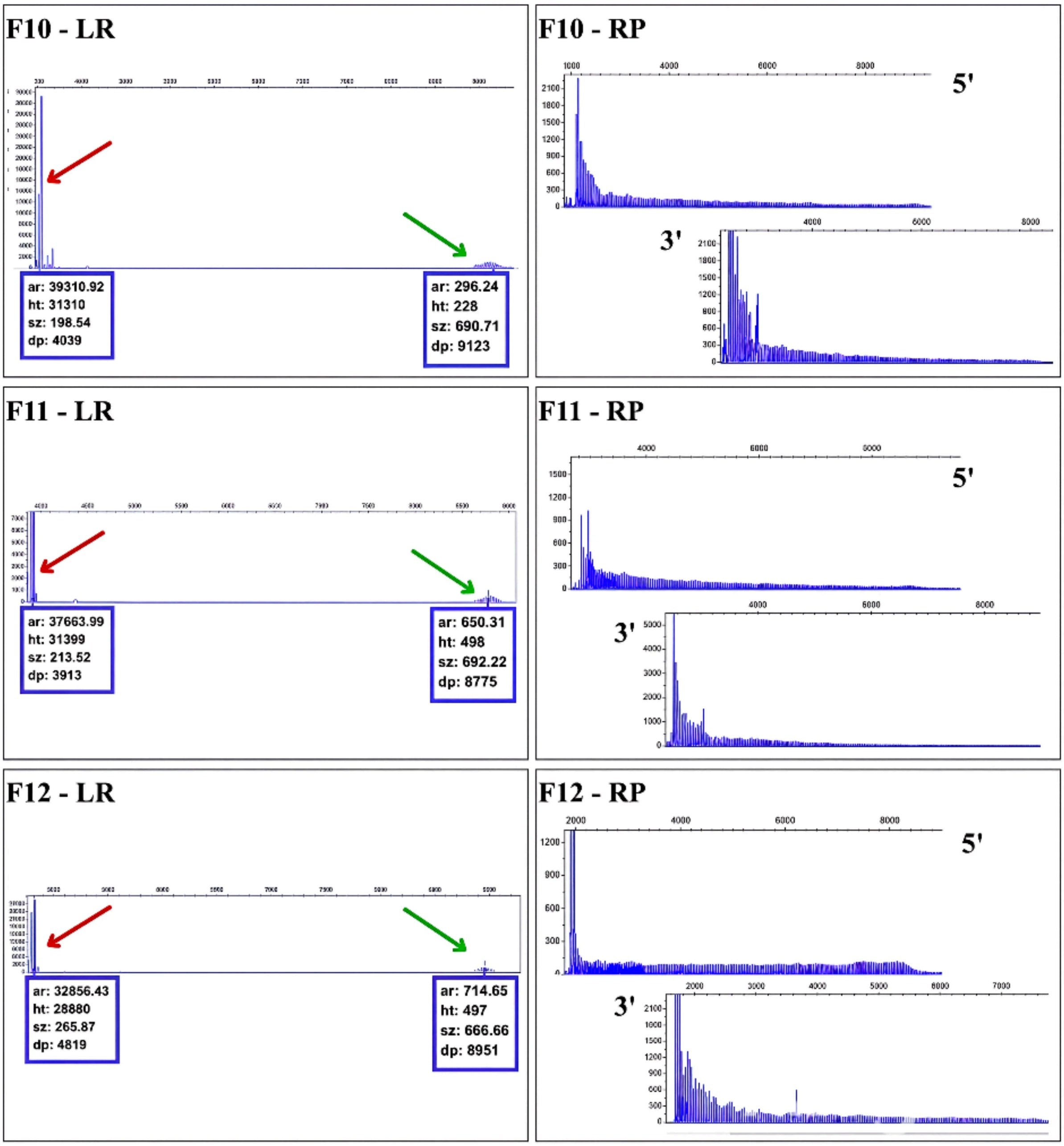

Supplemental Figure 1. Representative electropherograms of individuals with intermediate alleles confirmed as expansion-positive. The left panels (LR) show results from standard sizing (Long-Range PCR), where red arrows indicate the wild-type alleles and green arrows indicate the detected expanded/intermediate alleles. Note that while standard sizing suggests sizes within or near the intermediate range, the right panels (RP) provide confirmation of the repeat expansion. The 5' and 3' RP-PCR assays show the characteristic sawtooth appearance, confirming the presence of a pathogenic expansion in individuals F10, F11, and F12.

SUPPLEMENTAL TABLE 1. The primer sequences and experimental conditions

	Primers	Reagents
**Long-Range**	Fw: TGCAAATGAAGGAAAACTCTT<br>Rv: CAATGATGAATTAAGCAGTTCC	H2O - 3,5μL<br>Phushion Flash Master Mix 2X (Thermo Scientific - F548) - 5μL<br>Primers - 0,25μL<br>gDNA - 1μL
**Triplet-Primed 5'**	Fw: TGCAAATGAAGGAAAACTCTT	H2O - 3,48μL
	Rv: CACGACGTTGTAAAACGACTTCTTCTTCTTCTTCTTCTTC	Phushion Flash Master Mix 2X (Thermo Scientific - F548) - 5μL
	FAM: CACGACGTTGTAAAACGAC	P.Fw - 0,25μL
		P.Rv - 0,025μL
**Triplet-Primed 3'**	Fw: TGCCCACATAGAGCTTAGTCT	FAM - 0,25μL
	Rv: CACGACGTTGTAAAACGACGAAGAAGAAGAAGAAGAAGAA	gDNA - 1μL
	FAM: CACGACGTTGTAAAACGAC	
